# The central role of nucleic acids in the pathogenesis of systemic lupus erythematosus

**DOI:** 10.12688/f1000research.17959.1

**Published:** 2019-04-03

**Authors:** David S. Pisetsky

**Affiliations:** 1 Department of Medicine and Immunology, Duke University Medical Center and Medical Research Service, VA Medical Center, Durham, NC, USA

**Keywords:** Lupus, DNA, RNA, antinuclear antibody, interferon, DNase, microparticles

## Abstract

Systemic lupus erythematosus (SLE) is a prototypic autoimmune disease whose pathogenesis can be conceptualized by a model based on a central role for immune complexes (ICs) between antinuclear antibodies and nucleic acids. According to this model, ICs can promote pathogenesis by two main mechanisms: deposition in the tissue to incite local inflammation and interaction with cells of the innate immune system to stimulate the production of cytokines, most prominently type 1 interferon. The latter stimulation results from the uptake of DNA and RNA in the form of ICs into cells and subsequent signaling by internal nucleic acid sensors for DNA and RNA. These sensors are likely important for the response to intracellular infection, although they may also be triggered during cell stress or injury by DNA or RNA aberrantly present in the cytoplasm. For IC formation, a source of extracellular DNA and RNA is essential. The current model of SLE posits that cell death is the origin of the nucleic acids in the ICs and that impairment of clearance mechanisms increases the amount of nuclear material in the extracellular space. This model of SLE is important since it points to new approaches to therapy; agents targeting interferon or the interferon receptor are examples of therapeutic approaches derived from this model. Future studies will explore novel biomarkers to monitor the operation of these mechanisms and to elucidate other steps in pathogenesis that can be targeted for therapy.

## Introduction

Systemic lupus erythematosus (SLE) is a prototypic systemic autoimmune disease that primarily affects young women and causes highly variable clinical and serological manifestations ^1, 2^. Clinically, SLE is marked by inflammation and damage of multiple organ systems, including the joints, skin, kidney, nervous system, and blood. Immunologically, SLE is associated with the production of autoantibodies to a wide variety of macromolecules, especially those in the cell nucleus (antinuclear antibodies, or ANAs). The widespread autoreactivity in SLE has suggested a role of more generalized or global immune disturbances in pathogenesis. Indeed, studies on immune cell function and phenotype have identified a multitude of B- and T-cell disturbances that could promote autoreactivity ^3^.

An important clinical condition because of its major impact on young women, SLE has been widely studied as a model for immune regulation since its pathogenesis involves the most critical steps for immune tolerance. In tolerance, the recognition of self-antigens is prevented by a host of mechanisms operating in both B and T cells. In SLE, tolerance is breached and autoantibody expression occurs, driving inflammatory manifestations. The study of SLE pathogenesis is also important since effective treatment is limited at present and the development of new agents for SLE can provide a setting to gain fundamental new insights into immunosuppression relevant not only for SLE but also for other autoimmune diseases. This review will consider a current model for mechanisms underlying the pathogenesis of SLE and the implications for the development of new biomarkers and treatment.

## Model for systemic lupus erythematosus pathogenesis

SLE can affect many organ systems; individual patients, however, generally show more limited patterns of involvement. Indeed, SLE can vary from a relatively mild condition of skin and joints to a fulminant condition leading to rapidly progressive glomerulonephritis. Although the basis of this heterogeneity is unknown, certain features suggest the operation of some common mechanisms that encompass various patient subsets. Thus, patients with SLE almost always produce characteristic ANAs, and ANA expression is a criterion for disease classification ^4, 5^. In addition, genome-wide association studies have identified polymorphisms highly associated with SLE. In this construct, a variety of different genes, including some related to ancestry, may predispose patients to autoimmunity, and the array of genes in association with environmental exposures confer heterogeneity ^6^.

**Figure 1.**
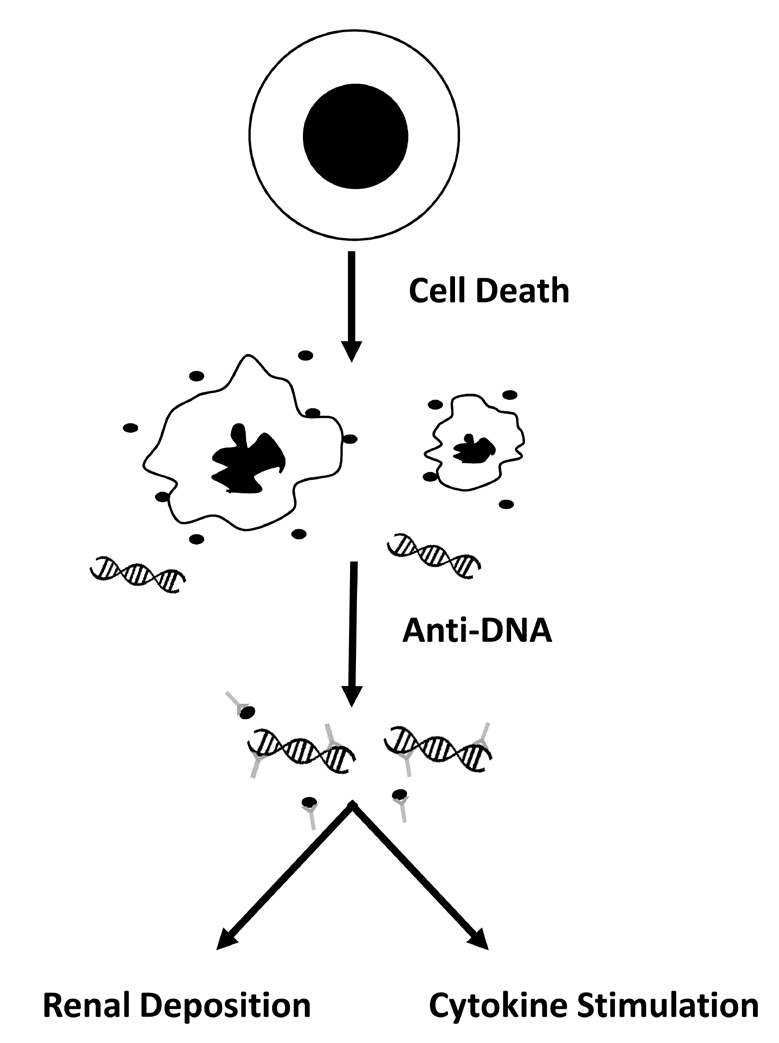
The role of DNA and anti-DNA in the pathogenesis of systemic lupus erythematosus. The figure provides a schema for the pathogenesis of systemic lupus erythematosus. In this model, as cells die by apoptosis, the nucleus collapses and fragments, and the cell body shrinks, producing apoptotic bodies as well as microparticles (filled circles) which contain DNA. In addition, the cell releases DNA in a free form; in the free form, DNA may be associated with histones to form the nucleosome. DNA in microparticles as well as free DNA can bind to anti-DNA antibodies to form immune complexes. These immune complexes then can deposit in the kidney to induce nephritis or can stimulate cytokine production following uptake by plasmacytoid dendritic cells. Thus, DNA can start on the inside of one cell, translocate to the outside in the blood, and then go back into the inside of another cell. A similar mechanism pertains to anti-RNA-binding protein (anti-RBP) antibodies, although, for these specificities, antibodies bind to the protein rather than the RNA.

In recent years, studies of patients with SLE and mouse models of lupus have produced a coherent, even compelling, model of disease to guide investigation and provide a framework for new therapy. This model is based on the proposition that, in SLE, aberrant responses to nucleic acids disrupt immune regulation and, in a genetically susceptible individual, drive ANA production. These ANAs in turn can form immune complexes (ICs) with nucleic acids that have entered the circulation as a consequence of cell death. Disease manifestations result from downstream actions of these ICs which have two distinct roles in disease. The first role is the deposition in the kidney to induce nephritis, an important determinant of morbidity and mortality. The second (and perhaps unexpected) role derives from the immunological actions of nucleic acids that become manifest when in the form of ICs ^7– 10^. Figure 1 illustrates this model.

## Role of antinuclear antibodies

In determining the immunological consequences of ICs containing nucleic acids, ANA specificity is key. In SLE, ANAs target highly conserved nuclear molecules that are present in all cells. These antibodies can be conveniently divided into families on the basis of the nucleic acid content ^5, 11, 12^. The first family is directed to components of the nucleosome and prominently includes anti-DNA. These antibodies bind to both single-stranded and double-stranded DNA and react to determinants present on the phosphodiester backbone ^13, 14^. In contrast, antibodies to RNA-binding proteins (RBPs) target complexes of RNA and proteins. These complexes are denoted as Sm, RNP, Ro, and La; in all cases, the antibodies bind to the protein and not the RNA ^5, 12^. Despite differences in the structure of their antigens and pattern of expression, anti-DNA and anti-Sm are both serological criteria for SLE.

The screening for ANAs has long been a central element in patient evaluation, although the role of specific ANAs in disease pathogenesis has until now been less certain. Anti-DNA has been the exception since, in SLE, there is clear evidence that ICs composed of anti-DNA antibodies deposit in the kidney; furthermore, in many patients, levels of anti-DNA rise and fall with disease activity, especially nephritis activity. In this situation, depression in complement levels can occur concordantly, pointing to the presence of ICs. Unlike those of anti-DNA, levels of anti-RBPs are frequently static during the course of disease, showing little change with disease activity. As such, it has been difficult to relate anti-RBPs to either disease activity or particular clinical manifestations ^5, 15^.

Recent studies, however, have provided a new picture of the role of ANAs and revealed a mechanism by which ICs with either anti-DNA or anti-RBPs can induce inflammation. This mechanism involves the stimulation of cells of the innate immune system, especially plasmacytoid dendritic cells, to produce type 1 interferon and other pro-inflammatory mediators. Type 1 interferons are an ensemble of cytokines that are pleiotropic in action and can promote many of the clinical and immunological features of SLE. With current technology, the presence of type 1 interferon is usually assessed by the analysis of the pattern of gene expression in peripheral blood cells rather than the immunochemical assay of interferon itself ^16– 18^. An “interferon signature” occurs prominently in many patients with SLE and has spurred the development of therapies that target either members of the interferon family or the interferon receptor ^19– 22^. In this regard, levels of interferon may relate to polymorphisms in genes encoding signaling proteins involved in transcriptional control of interferon as well as a complex interplay between the nature of the ICs and regulations of interferon expression ^23– 25^.

## Nucleic acid sensors

The reason that ICs with DNA and RNA can drive cytokine production relates to the intrinsic immunological properties of nucleic acids. Indeed, DNA and RNA both have potent immunostimulatory activity; depending on the source, DNA and RNA can serve as PAMPs (pathogen-associated molecular patterns) or DAMPs (damage- or death-associated molecular patterns) to activate innate immunity via internal nucleic acid sensors. These sensors include Toll-like receptor (TLR) 3, 7/8, and 9 as well as non-TLR sensors ^26^. Non-TLRs include the nucleotide-binding and oligomerization domain (NOD) receptors (or NLRs) and the retinoic acid–inducible gene 1 (RIG-1)-like receptors (RLRs). Importantly, these sensors reside on the inside of cells in contrast to other sensors or pattern recognition receptors (PRRs) which are present on the outer cell membrane (for example, TLR 4 for lipopolysaccharide, or LPS).

As shown in *in vivo* and *in vitro* systems, internal nucleic acid sensors play a key role in host defense against intracellular infection by viruses and bacteria. Furthermore, internal sensors can mediate the response to events such as oxidative stress. In these situations, nucleic acids translocate from their usual location or compartment and gain access to internal sensors to activate inflammation. Among organelles, mitochondria represent an abundant source of DNA that can access internal sensors. Compared with nuclear DNA, mitochondrial DNA is more potent immunologically because of its base sequences and content of oxidized bases. Perhaps reflecting the origin of mitochondria as symbiotic bacteria, mitochondrial DNA resembles bacterial DNA in its content of CpG motifs (cytosine guanosine dinucleotides) which confer PAMP activity on foreign DNA ^27, 28^. Table 1 lists determinants of the immune properties of DNA.

**Table 1.** Determinants of immune properties of DNA.

Sequence Size Backbone structure Oxidation state Source (that is, nucleus versus mitochondria) Protein binding Presence in an immune complex Intracellular location

While RNA is present abundantly in the cytoplasm of cells, stimulation of RNA sensors occurs with particular forms of these molecules or the interaction of sensors with RNA in particular locations ^29^. Thus, stimulation of the RIG-1 sensor occurs with RNA from certain viruses that display characteristic structural features at the 5′ end of the RNA molecule. For the MDA5 sensor, stimulation may depend on the length and structure of the RNA molecule, including long stretches with base pairs ^30^. For TLRs recognizing RNA, the interaction occurs in an endosomal compartment, although transport systems can allow movement of RNA from the endosomal compartment to the cytoplasm for interaction with RLRs ^31^. Another source of stimulatory RNA is mitochondrial RNA that has entered the cytoplasm because of a deficiency of enzymes involved in degradation ^32^.

Among internal sensing systems, the cyclic GMP-AMP synthase–stimulator of interferon genes (cGAS-STING) pathway can mediate the response to cytosolic DNA and has attracted interest as a target of therapy in diseases such as cancer and autoimmunity ^33, 34^. In this response, the protein cGAS binds to DNA to catalyze the reaction of GTP and ATP to form cyclic GMP-AMP (cGAMP). cGAMP in turn binds to STING that leads to the phosphorylation of IRF3, which induces the transcription of pro-inflammatory genes. Although this pathway may be important in host defense to DNA viruses, it can be activated during cellular stress when DNA from the nucleus or mitochondria translocates to the cytoplasm. Agents targeting the cGAS-STING system are in development as novel therapies for SLE ^35^.

Along with systems that sense cytoplasmic nucleic acids are systems that degrade DNA and RNA that have gained access to the cytoplasm. These putatively protective systems include nucleases such as TREX, which is a 3′ exonuclease that can degrade double-stranded DNA. In humans, mutations in TREX are associated with Aicardi–Goutières syndrome (AGS); AGS has some features of SLE as well as prominent production of type 1 interferon, especially in the central nervous system ^36, 37^. Mice lacking TREX also develop features of SLE, including myocarditis, suggesting that an increase in intracellular DNA can drive disease by triggering internal DNA sensors. This possibility is supported by observations that mice with a double knockout of TREX and cGAS are protected from autoantibody production as well as tissue inflammation ^38, 39^.

Mutations in internal nucleic acid sensors and nucleases are rare causes of SLE in humans, although these conditions suggest a mechanism by which nucleic acids can drive autoreactivity via the internal receptors. In this schema, ANAs are critical effectors since they can form ICs that transport DNA and RNA into cells of the innate immune system. Once inside the cell, nucleic acids can interact with sensing systems to stimulate interferon and other cytokines ^40– 43^. Since there are sensors for DNA and RNA, ICs from both anti-DNA and anti-RBP antibodies can activate this pathway, accounting for the association of these ANAs with the interferon signature. Importantly, the interferon signature does not vary much with disease activity, suggesting that anti-RBP antibodies may be prominent players in this response because of their high titers and chronic expression ^4, 15, 44– 46^.

Although ANAs are an important sign of autoimmunity in lupus, their presence may not be sufficient to induce disease manifestations. Indeed, ANA expression can predate clinical disease manifestations by many years in a state called pre-autoimmunity ^47– 49^. Other elements must be present to convert the serological manifestations into clinical manifestations. Among these elements, self-antigen, present in quantities sufficient to allow IC formation, may be key. In this conceptualization, the development of disease is a two-step process. The first step is ANA production. This step may occur in genetically susceptible individuals who, following an infection, for example, produce a cross-reactive antibody that binds both a foreign and a self-antigen; genetically determined disturbances in B- and T-cell regulation may underlie a tendency to produce cross-reactive antibodies and breach tolerance.

## Generation of extracellular nuclear antigens

While ANAs may be pathological (that is, aberrantly produced), alone these antibodies may not be pathogenic (that is, cause specific disease manifestations). In IC disease, pathogenicity is multifactorial and depends on the immunochemical properties of antibodies such as avidity and fine specificity as well as the availability of self-antigen. Although, by definition, self-antigen is always present in the organism, it may not be present in sufficient concentrations or locations to form ICs to induce nephritis or drive cytokine production. The most likely source of self-antigen for IC formation is cell death since every day a large number of cells die and can release their contents.

Since DNA and other endogenous molecules (DAMPs) arising during death are potentially immunostimulatory, dying cells and their “dangerous” contents must be removed in a safe or silent way. In addition to the role of phagocytosis, clearance depends on serum proteins such as complement, DNases, and RNases to manage the load of nucleic acids that could fill the blood ^50^. Interestingly, deficiency of C1q, a complement component important for clearance, represents a single gene model for SLE, suggesting that increased levels of both intracellular and extracellular nucleic acids are pathogenic.

As now recognized, cells die by a variety of biochemically and morphologically defined forms of death which depend on the nature of the inducing stimulus and the cell type. Among these death forms, apoptosis occurs in both physiological and pathological settings and involves the systematic disassembly of the cell mediated by enzymes known as caspases. During apoptosis, DNA along with other nuclear molecules is cleaved and rearranged, possibly to reduce immune activity. Furthermore, as apoptosis proceeds, nuclear molecules can translocate to the cell membrane to enter blebs. Although the function of blebs is not well understood, the localization of nuclear antigens in these structures may alter their immunological properties. Given the frequency of cell death in the body, apoptosis is often considered the death form that creates extracellular DNA and RNA ^51, 52^.

Apoptosis is not the only death form that can release DNA, however, since necrosis, a form of accidental cell death, can also increase levels of extracellular DNA presumably because of cell lysis and destruction that characterize this process. In contrast to necrosis, necroptosis is a form of programmed cell death that can be induced by a variety of agents and involves the activity of enzymes known as the receptor-interacting protein kinases. Necroptosis can occur with the inhibition of caspases during stimulation by TLR agonists, suggesting complex regulatory interactions that can lead to death or activation ^53^. Importantly, cells undergoing necroptosis can release nuclear molecules and other cellular constituents with immune activity ^54, 55^.

A particularly novel death process that occurs primarily with neutrophils is termed NETosis ^56, 57^. NETosis can be induced by a variety of stimuli and is associated with the breakdown of the nuclear membrane, the mixing of DNA with the granule enzymes, and the extracellular release of a mesh-like structure known as a NET (neutrophil extracellular trap). A NET can entrap bacteria for killing by the protein components such as myeloperoxidase and histones; a NET can also contain mitochondrial DNA. In addition to having an anti-bacterial function, a NET can damage endothelium and serve as a source of DNA for IC formation ^58^. Thus, an increase in the concentration of DNA in the blood can occur by a number of mechanisms that operate in SLE.

Although DNA in the blood can exist in a free form (albeit attached to histones), it may also be present as a component of microparticles (MPs). MPs are extracellular vesicles that can be released from activated as well as dead and dying cells, possibly representing blebs that have detached from cells undergoing apoptosis ^59^. These subcellular structures are approximately 0.1 to 1.0 microns in diameter and contain a range of membrane, cytoplasmic, and nuclear molecules, including DNA ( Table 2). Importantly, the DNA in MPs can be bound by anti-DNA antibodies either because it resides on the particle surface or because the particle is sufficiently porous to allow the entry of antibodies into its interior. As shown with murine monoclonal anti-nucleosomal antibodies, only some anti-DNA can bind particles, suggesting that the display of DNA epitopes may be selective ^60, 61^.

**Table 2.** Properties of microparticles.

Membrane-bound vesicles 0.1 to 1.0 microns Ensemble of nuclear, cytoplasmic, and membrane molecules Source of extracellular nucleic acids Formation of immune complexes with antinuclear antibodies

Studies using flow cytometry demonstrate that the blood of patients with SLE contains increased numbers of particles with bound IgG as well as complement. Furthermore, the levels of the IgG-positive particles may be related to levels of anti-DNA as well as disease activity ^62– 66^. There is also evidence that ICs containing particles can deposit in the renal glomerulus, as shown by histopathologic staining of kidney biopsies for the galectin-3-binding protein, a component of MPs ^67^. Together, these findings point to MPs as an important source of DNA for IC formation. Although ICs have long been considered an essential element in disease pathogenesis, their physical identification using conventional approaches appropriate for analyzing soluble complexes has, in fact, been difficult. The formation of ICs based on a particle structure would be fundamentally different from that of a complex with a soluble antigen and would require other analytic techniques for identification and quantification, including the use of plasma.

As discussed above, the amount of DNA in the blood depends on processes that increase its concentration (that is, cell death) and the processes that decrease its concentration (that is, nucleases). Although the enzyme DNase 1 can digest DNA, studies on a related DNase called DNase 1-like 3 (DNase 1L3) have provided a new perspective on the degradative process ^68, 69^. DNase 1L3 differs in its specificity for DNA from DNase 1. Whereas DNase 1 degrades free DNA, DNase 1L3 degrades DNA in the form of nucleosomes, likely an important form of extracellular DNA. Patients with mutations in DNase 1L3 present with a vasculitis-like condition, whereas mice with a knockout of the gene for DNase 1L3 develop a lupus-like illness with anti-DNA production and a dramatic increase in DNA associated with MPs ^70– 74^. These observations provide evidence for the relevance of particles as a source of extracellular DNA for IC formation and suggest the use of MP-ICs as biomarkers.

## Unanswered questions

This model for pathogenesis of SLE is very plausible and has considerable support from experimental data. Nevertheless, many aspects are unknown. Salient issues that can be the subject of future study concern the actual form of DNA (and other nuclear molecules) in the extracellular milieu, the specificity of ANAs that can form ICs, and the detailed mechanisms for the trafficking of DNA and RNA molecules that are introduced into the inside of cells in the form of ICs. In terms of host defense, events on the inside of cells are becoming as important as events outside of cells. Understanding of events in SLE will therefore need fundamental investigation to elucidate the role of immune signaling by cytoplasmic nucleic acids in the context of infection.

## Conclusions

Historically, SLE has been conceptualized as a disease of ICs composed of ANAs and their cognate nuclear antigens. Although this basic schema still pertains, recent studies have provided a new and unexpected picture of the basic triad of autoantigens, autoantibodies, and ICs by demonstrating that (1) nucleic acids are immunologically active, (2) nucleic acids can trigger receptors that are part of an internal host defense system, and (3) ICs may involve large antigenic structures in which DNA and RNA are embedded in particles. Furthermore, recent studies have delineated systems that regulate the levels of both intra- and extra-cellular nucleic acids and thereby their ability to drive inflammation. In this model, DNA or RNA can leave one cell and, via ICs, enter another to trigger an internal receptor. Future studies will translate this basic information into the creation of new biomarkers and the development of new therapies that can target more specifically the pathways by which nucleic acids initiate and sustain autoreactivity.
